# Elevational Synthetic Aperture Focusing for Rotated Array-Based Three-Dimensional Ultrasound Imaging

**DOI:** 10.1109/access.2025.3549638

**Published:** 2025-03-10

**Authors:** RYO MURAKAMI, YANG WANG, YICHUAN TANG, RYOSUKE TSUMURA, GREGORY S. FISCHER, HAICHONG K. ZHANG

**Affiliations:** 1 Department of Robotics Engineering, Worcester Polytechnic Institute, Worcester, MA 01609, USA; 2 Department of Biomedical Engineering, Worcester Polytechnic Institute, Worcester, MA 01609, USA; 3 Department of Computer Science Engineering, Worcester Polytechnic Institute, Worcester, MA 01609, USA

**Keywords:** Biomedical imaging, focusing, image processing, medical diagnostic imaging, ultrasonics imaging

## Abstract

Three-dimensional (3D) ultrasound (US) imaging is widely used for real-time, non-ionizing, and cost-effective medical diagnostics. However, using a one-dimensional (1D) transducer often results in limited elevational resolution due to the inherent beam thickness. In this paper, we introduce an elevational Synthetic Aperture Focusing (SAF) algorithm specifically designed for rotational 3D US imaging. Unlike previous methods requiring channel data, our approach operates on in-plane beamformed radio-frequency (RF) data, making it more accessible on many commercial scanners. Through simulations and experiments, we demonstrate significant improvements in elevational resolution (up to 96.4%) and contrast (up to 274.7%). These findings highlight the potential of the proposed algorithm to enhance both research and clinical applications of rotational 3D US imaging.

## I. INTRODUCTION

Ultrasound (US) imaging is renowned for its safety, non-ionizing characteristics, real-time imaging capabilities, and cost-effectiveness [1]. Although two-dimensional (2D) imaging is the most common form of US imaging, three-dimensional (3D) US imaging has been developed to provide more comprehensive and intuitive information for medical diagnosis and intervention [2], [3], [4], [5]. To create a 3D US image, a typical method involves capturing multiple imaging slices by mechanically moving a one-dimensional (1D) transducer array and accurately positioning these slices [5], [6]. Despite the need for probe manipulation and position tracking, this method offers advantages, such as easier manufacturing and high accessibility compared to 2D transducer arrays [7], which is also commonly used to obtain 3D US images without probe manipulation. However, a challenge with this mechanical scanning approach with a 1D transducer array is the inevitable elevational beam thickness due to the transducer array’s physical properties, which degrades resolution in that direction. To address this issue, the Synthetic Aperture Focusing (SAF) algorithm has been used in various 3D scanning methods, including sweeping [8] and linear translation with reflectors [9]. The SAF is, in general, a technique to virtually enlarge the imaging aperture to enhance the image quality [10], [11]. Specifically, in the context of the elevational SAF for 1D array transducer, we can assume the elevational focal point as the virtual source of transmission because the wave-front above and below it can be seen as a circular wave from the focal point in a certain angle [12]. In other words, each 2D image should carry a certain amount of information along the elevational direction, and the core idea of the elevational SAF is to combine these 2D slices acquired at different positions and perform beamforming along the elevational direction. Thus, the SAF’s advantages include: 1) enhancing resolution and contrast, while the disadvantages include: 1) higher computational load. Rotational 3D US imaging, namely rotating a 1D array probe, is another scanning configuration that allows for acquiring 3D US images within confined spaces [13]. This rotation configuration offers advantages over linear or sweeping motions in that it does not require additional geometrical space to allow the probe to be manipulated. As a relevant concept to this paper, Nikolov et al. [14], [15] introduced the application of the SAF algorithm for rotational 3D US, aiming at achieving a high frame rate without compromising the image quality of 3D US images. They employed a phased array transducer with continuous rotation and transmitted acoustic waves in a sparse way to boost the frame rate [16]. With the unique transmit and receive sequence, they achieved a frame rate of 10 volumes per second while maintaining the comparable spatial image resolution to the original phased array [14]. However, implementing the transmit and receive sequence proposed in this work requires a programmable US controller at a single element level and access to the channel data of each element. Given the fact that most of the existing US systems in the market do not allow such specific programming and data access, the proposed SAF algorithm would have difficulties being widely used in either research or clinical environments. For wider use of the SAF algorithm for rotational 3D US, it is critical to make the algorithm compatible with more accessible US data. In addition, the validation is limited in evaluating target image quality placed at specific geometrical locations and is not generalized. Thus, our primary objective in this study is to develop an SAF algorithm for rotational 3D US imaging with high accessibility.

Here, we propose an SAF algorithm for rotational 3D US imaging that improves image qualities such as resolution and contrast. The proposed SAF algorithm takes in-plane beam-formed radio frequency (RF) data as input, which is accessible in many scanners [17]. In addition, considering the unique geometrical constraints associated with the rotational SAF, we study the image quality variation due to the variable target location distribution and conditions. The primary novelty of this work lies in formulating an SAF algorithm under these requirements. Our contributions made in this study can be summarized as follows: 1) we first formulated the elevational SAF algorithm based on post-beamformed in-plane RF data for the rotational 3D US to improve image resolution and contrast with its performance evaluation, and 2) we evaluated the influence of the target’s geometrical variation such as the depth and the spacial position with respect to the array on the rotational SAF performance. These contributions are expected to boost the potential of SAF in enhancing image quality and accessibility for both research and clinical environments, which should result in accelerating research and better diagnosis/treatment.

The rest of this paper is structured as follows: In Section II, we first formulate the SAF algorithm and define simulation environments and the experimental setups. Then, the results are provided followed by discussions and conclusions.

## II. MATERIALS AND METHODS

### A. SYNTHETIC APERTURE FOCUSING FOR ROTATIONAL 3D ULTRASOUND

**FIGURE 1.**
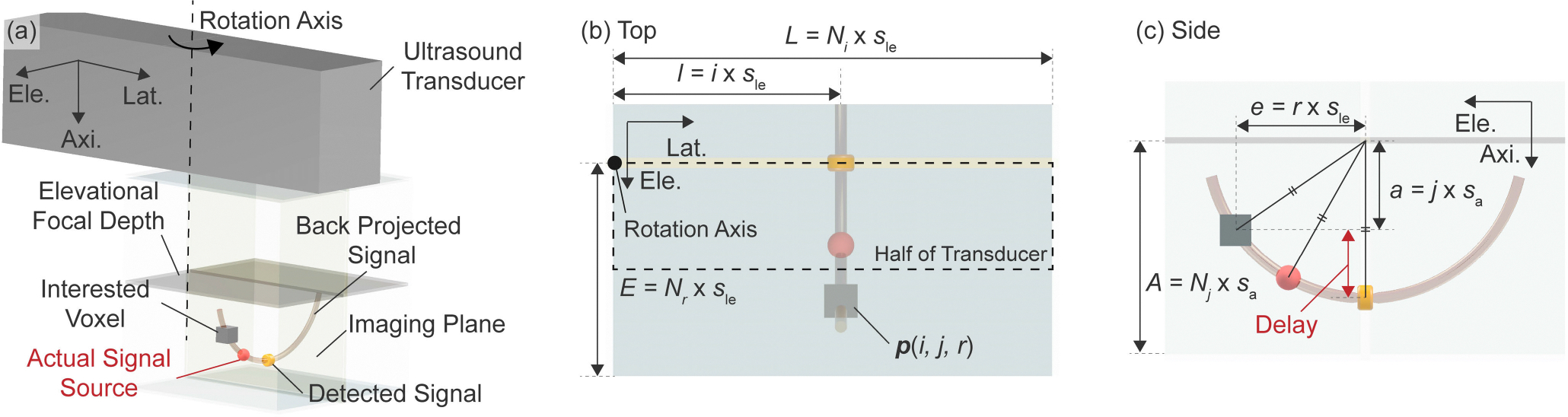
Schematics showing the concept of the proposed Synthetic Aperture Focusing Algorithm. (a) Bird’s eye view, (b) Top view, (c) Side view; The lateral half of the acquired ultrasound signal is used for back projection. The detected signal on the imaging plane is back-projected along the elevational axis at the angle the arc representing the back-projected signal will be vertically flipped between the upper and the lower region from the focal depth.

**FIGURE 2.**
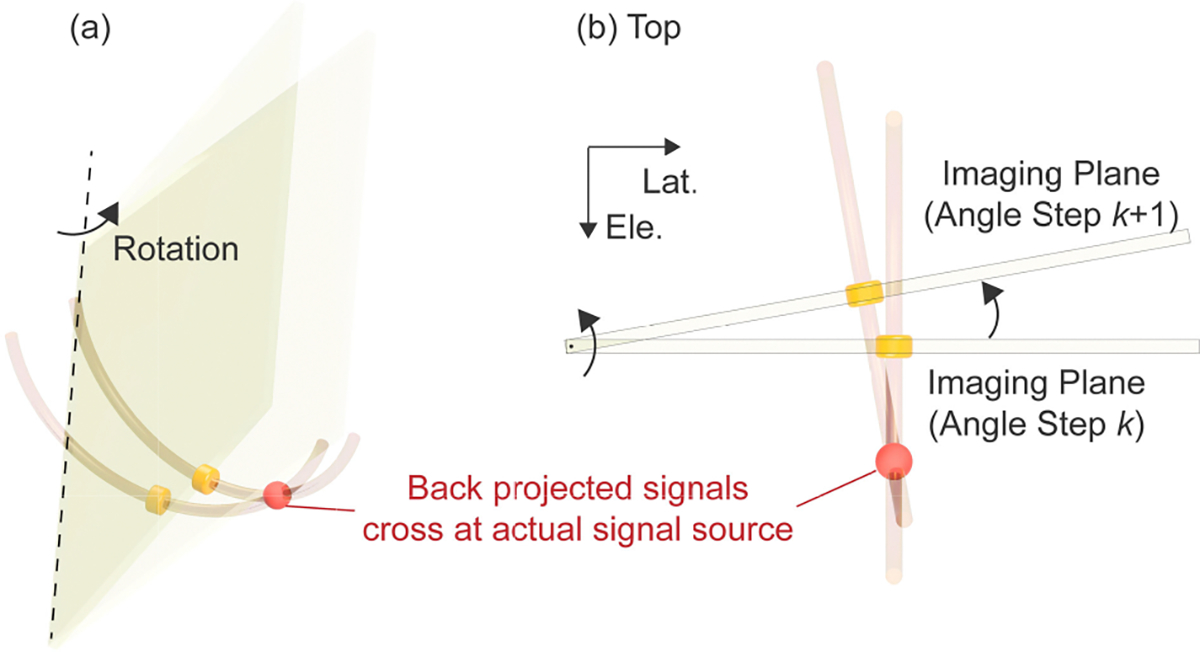
Schematics representing the idea of the back-projection-based signal enhancement. (a) Bird’s eye view, (b) Top view; The back-projected signals as arcs are expected to intersect in the 3D space at the location of the actual signal source and provide enhanced intensity after the summation.

In this study, the SAF algorithm is implemented based on the signal back-projection approach (Figure 1). It is assumed a 1D transducer array is rotated around the axial axis penetrating the center of the transducer by 180 degrees and that the 1D transducer would receive a certain amount of signal from a signal source even if it’s not at the right below the transducer due to the elevational beam thickness. The detected signal on the imaging plane is attributed to the corresponding signal source existing along the elevational direction. Although the 1D transducer array can not identify the exact axial distance to the signal source, it is possible to generate the theoretically expected locations of the signal source as a 1D path. The SAF algorithm generates the path, as an arc, by back-projecting the detected signal from the imaging plane. The theoretical time-of-flight (TOF) of the acoustic wave is considered in the back-projection process and the time delay results in the arc shape. This process provides one 3D volume data involving the possible signal source locations for each imaging slice. The SAF algorithm then sums up all the 3D volume data considering the geometrical relationships among the imaging planes. The summation is expected to highlight the locations of the actual signal sources because the arcs, the possible signal source locations, should overlap in the summation process at the actual signal source locations (Figure 2). In the implementation of the SAF algorithm, the transducer is divided into half along the lateral direction as illustrated in Figure 1 (a).

The formulation of the SAF algorithm is described in the rest of this section. The following formulation considers the region below the elevational focal depth, and the obtained formulation can be extended into the region above the elevational focal depth just by flipping the formulation with respect to the lateral-elevational plane. Note that, in this flipping process, the differences in the beam characteristics should be considered, and the parameters, such as the f-number, need to be tuned appropriately. Since the acoustic wave is focused at the elevational focal depth, the length along the axial direction is defined with respect to the elevational focal depth in the SAF algorithm. To implement the idea of the back projection, an imaging slice which is obtained as 2D data is expanded into 3D data (light blue box in Figure 1). Let the back-projected signal at the interested voxel be $S_{\text{back}}(l,a,e,\theta )$ and the corresponding detected signal on the imaging slice be $S_{\text{detect}}(l,a,\theta )$. Here l,a, and e represent the position along the lateral, axial, and elevational directions, respectively. θ denotes the rotation angle of the transducer. Since the imaging slice is defined on the imaging plane, the elevational position is always zero and omitted in the notation for $S_{\text{detect}}$. The relationship between the two variables can be represented as follows (Equation 1) considering the geometry illustrated in Figure 1 (c).

(1) $$S_{back}(l,a,e,\theta )=S_{detect}\left(l,\sqrt{e^{2}+a^{2}},\theta \right)$$

Equation 1 represents how the detected signal on the imaging plane is back-projected to the 3D space. Here, the maximum of e is regulated under the aperture growth mechanism. The remaining step to obtain the 3D data with SAF algorithm $B_{\text{SAF}}$ is summing up the back-projected signals considering the rotation of the transducer (Equation 2).

(2) $$B_{SAF}=\;\int_{0}^{2\pi }\;\int_{0}^{L}\;\int_{0}^{A}\;\;\;\times \int_{-E}^{E}\;R_{z}\left(\theta \right)P_{s}\left(l,a,e,\theta \right)de\;da\;dl\;d\theta$$

(3) $$P_{s}(l,a,e,\theta )=p(l,a,e)S_{back}(l,a,e,\theta )f_{apo}(e)f_{ag}(a,e)l$$

(4) $$R_{z}(\theta )=\left[\begin{array}{ccc} cos\;\theta & -sin\;\theta & 0\\ sin\;\theta & cos\;\theta & 0\\ 0 & 0 & 1 \end{array}\right]$$

where, $f_{\text{apo}},\;f_{\text{ag}}$ are the multipliers for the apodization, and aperture growth, respectively. Note that the apodization and aperture growth in the elevational direction are applied in the post-processing stage through software. p(l,a,e) is the position vector containing the corresponding back-projected signal $S_{\text{back}}$. L,A, and E are the lengths of the expanded 3D box along the lateral, axial, and elevational directions, respectively. In Equation 3, l is applied as another multiplier to compensate for the discrepancies in the number of imaging slices between points near the rotation center and the points far from the rotation center. Since the back-projected signal should be symmetry with respect to the imaging plane, the integration range for the elevational direction is defined as -E to E. Note that the location determined by l,a, and e is defined with respect to the rotation center to apply the rotation matrix $R_{z}(\theta )$. In this entire study, the SAF algorithm is implemented on the MATLAB platform (MATLAB, The MathWorks, Inc., Massachusetts, United States).

### B. TRANSDUCER SPECIFICATIONS

The specifications of the transducer used in both simulation and experiment are summarized in Table 1. Here, some of the variables used in Equations 1 and 2 are defined using the parameters in Table 1.

Let i,j, and r be the indices for the lateral, axial, and elevational directions, respectively and $N_{i},\;N_{j}$, and $N_{r}$ are the number of elements in each direction. Then, the following relationships can be derived:

(5) $$L=N_{i}\times s_{le}$$

(6) $$A=N_{j}\times s_{a}$$

(7) $$E=N_{r}\times s_{le}$$

(8) $$l=i\times s_{le}$$

(9) $$a=j\times s_{a}$$

(10) $$e=r\times s_{le}$$

where $s_{le}$ is the length per pixel index along the lateral and elevational directions (Unit: [mm/pix]) and $s_{a}$ is the one for the axial direction, respectively. Based on table 1, the following relationships can be found:

(11) $$s_{le}=p$$

(12) $$s_{a}=\frac{c\;\cdot \;10^{3}}{2f_{s}}$$

(13) $$N_{i}=N_{r}=N$$

### C. SIMULATION ENVIRONMENT

#### 1) TRANSDUCER CONFIGURATION

**FIGURE 3.**
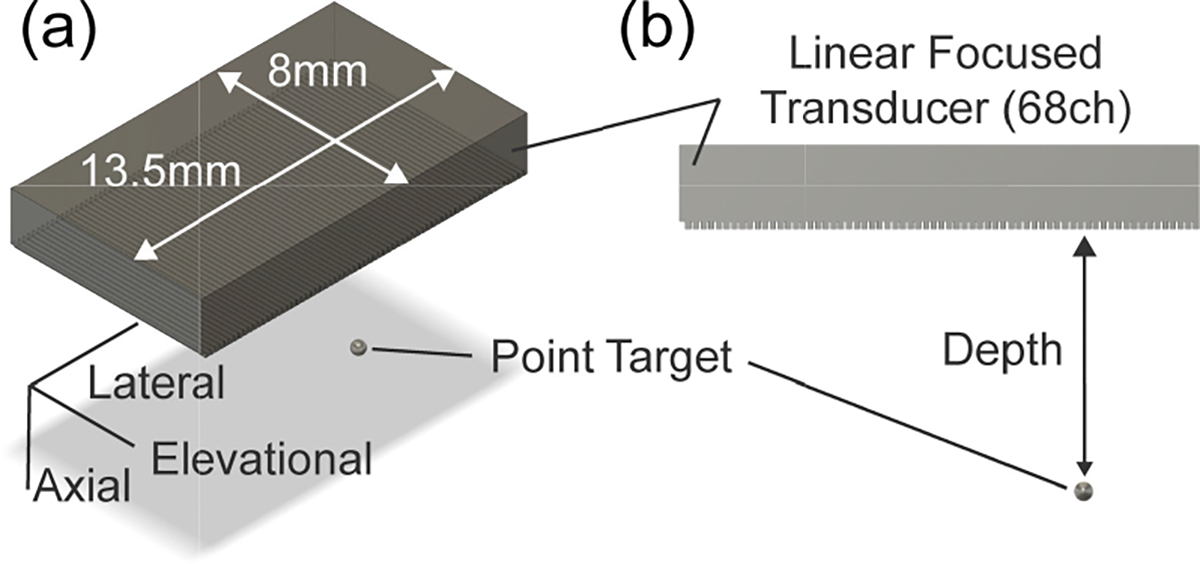
Drawings showing the simulation environment in the Field II software involving the linear array transducer and point target, (a) Bird’s-eye view, (b) Side view.

**FIGURE 4.**
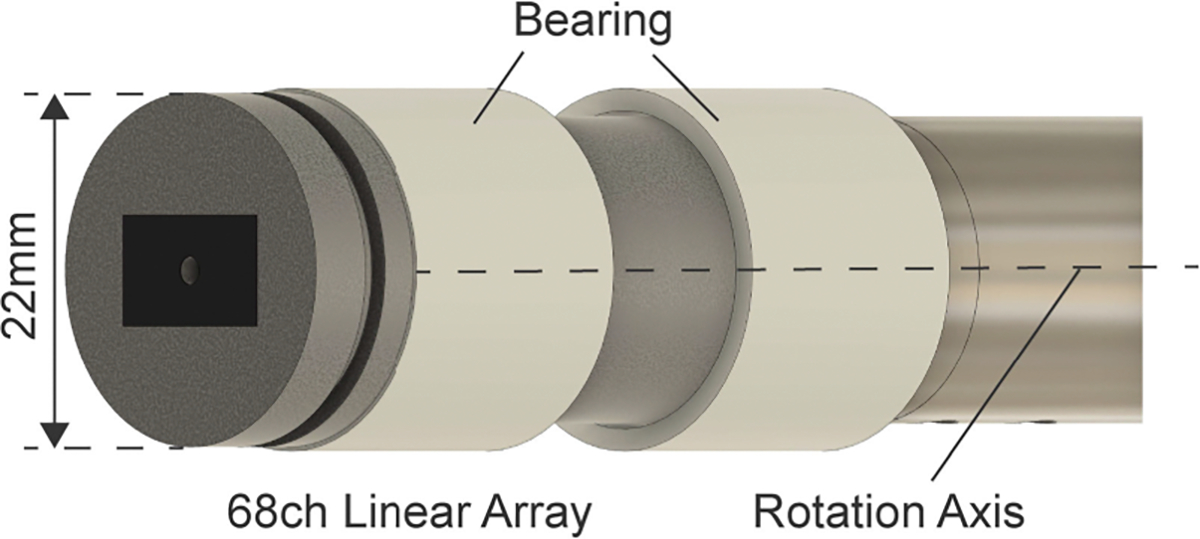
1D transducer array probe for rotational 3D US imaging [20].

The simulation is implemented using Field II simulation software [18], [19]. The probe we use in the experiments is simulated, and the probe’s design and parameters are summarized in Figure 4 and Table 1, respectively. To simulate the elevational beam thickness, the mode for an elevation-focused linear array (function: xdc_focused_array) is defined based on Table 1. Note that the transducer is fixed in the simulation environment and the scatter is moved to simulate the rotational motion. Figure 3 (a) shows the bird’s-eye view of the configuration for the simulation. The same elevation-focused linear array transducer is used to simulate both the transmission and reception of the acoustic wave.

#### 2) POINT TARGET

For the evaluation of elevational imaging resolution, a single scatter, as a point target, is placed below the transducer at several depths and distances from the center of rotation (Figure 3 (b), side view).

#### 3) CYST TARGET

To evaluate the algorithm’s performance in contrast, cysts are simulated in the same simulation environment. For the comprehensive analysis, the cyst size (Diameter: 6, 8, and 10 mm), the cyst depth (20, 30, and 60 mm), and the distance from the rotation center (1, 3, and 5 mm) are considered. The cyst is modeled as a sphere and simulations are conducted 10 times for each condition. In the simulation, a 20 × 20 × 20 mm space is defined around the target cyst, within which a total number of 10,000 scatterers are distributed using a uniform random distribution. The amplitude of each scatterer was assigned a value between 0 and 1 based on a normal random distribution. Finally, the amplitudes of the scatterers located within the cyst region are set to 0.

### D. EXPERIMENTAL SETUP

#### 1) ULTRASOUND IMAGING SETUP

The customized linear array transducer (68-element linear array transducer (element pitch: 0.2 mm), Japan Probe Co, Ltd., Kanagawa, Japan) is used in this study (Figure 4), which was developed and reported in our previous study [20]. In order for the probe to rotate, two glass-filled PTFE bearings (iglide^®^ i3-PL, igus, Inc., Rhode Island, United States) are installed. For US signal reception, a US data acquisition system (Vantage 128, Verasonics, Kirkland, WA, United States) was used and the IQ data was recorded in scanning.

#### 2) ACTUATION MODULE

For the rotation of the probe, an actuation module was also developed in the previous study [20]. The actuation module consists of the motors (USR30-S4N, SHINSEI Corporation, Tokyo, Japan), their motor drivers (D6030/24V, SHINSEI Corporation, Tokyo, Japan), motor controller (DMC-4143-CARD, Galil Motion Control, Inc., Rocklin, CA, United States), and optical encoders (E2–1250-157-IE-H-D-1, US Digital Corporation, WA, United States). The rotation of the motors is transferred to the probe through the timing belts. Note that, only one motor is used in this study given its experiment design despite the module being capable of actuating two axes independently. The encoder-based angular feedback control is implemented in the MATLAB environment (MATLAB, The MathWorks, Inc., Massachusetts, United States). Throughout this study including both simulations and experiments, the rotation of the probe is performed with 1-degree steps and the data from 0 degrees to 179 degrees are used for the 3D image generation.

#### 3) POINT TARGET PHANTOM

A bead sphere is used to evaluate the SAF algorithm’s performance in terms of the resolution. The point target phantom consists of a black metal sphere (Diameter: 1 mm) placed at a certain depth from the transducer. The sphere is fixed in a transparent gel wax, which is attached to the linear stage so that only the depth of the target can be modulated. The probe, actuation module, and the point target phantom with the linear stage are submerged in water to achieve the acoustic coupling.

#### 4) BREAST PHANTOM

**FIGURE 5.**
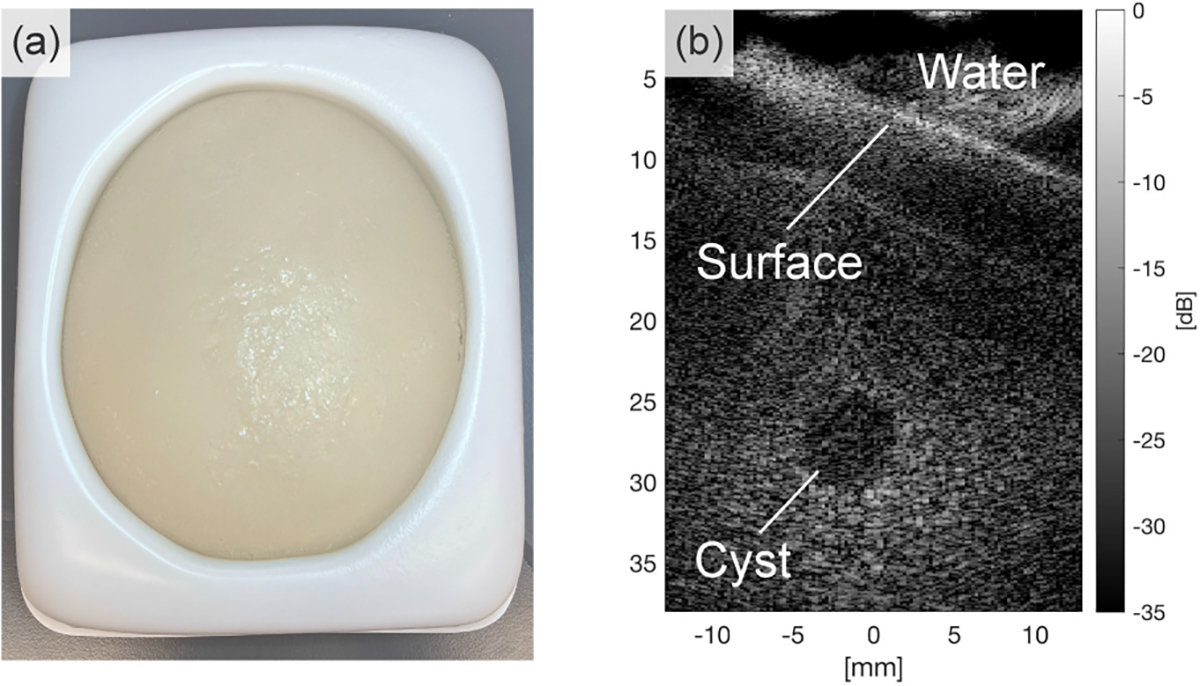
Breast phantom; (a) Photo from the top, (b) B-mode ultrasound image capturing the targeted cyst.

For the experimental performance evaluation of the SAF algorithm in cyst imaging, a breast phantom containing simulated cysts is used (Figure 5 (a) and (b)) (Part Number: 1552–01, CIRS Inc., Norfolk, VA, United States).

### E. EVALUATION METRICS

Two metrics are selected to evaluate the performance of the SAF algorithm in the aspects of resolution and contrast.

#### 1) FULL WIDTH AT HALF MAXIMUM (FWHM)

Resolution with/without the SAF algorithm is evaluated based on the FWHM [21]. Since the elevational resolution needs to be evaluated considering the rotational motion of the transducer, the maximum intensity projection (MIP) images are generated and each intensity profile is sampled as a circular path. MIP display is one of the methods to compress 3D images into 2D by projecting the maximum value of pixels along the projection path.

#### 2) CONTRAST-TO-NOISE RATIO (CNR)

The performance of the SAF algorithm for cyst imaging is evaluated using CNR, which is regarded as the index equivalent to lesion detectability [22]. CNR is calculated as follows:

(14) $$CNR=\frac{\left|\mu_{\text{target}}\;-\;\mu_{back}\right|}{\sqrt{\sigma_{\text{target}}^{2}\;+\;\sigma_{\text{back}}^{2}}}$$

$\mu_{\text{target}}$ and $\mu_{\text{back}}$ denote the average image intensities in a small area within the target and in the adjacent background, respectively, while $\sigma_{\text{target}}$ and $\sigma_{\text{back}}$ are the respective variances for these regions [23].

## III. RESULTS

### A. SIMULATION STUDY

#### 1) POINT TARGET

**FIGURE 6.**
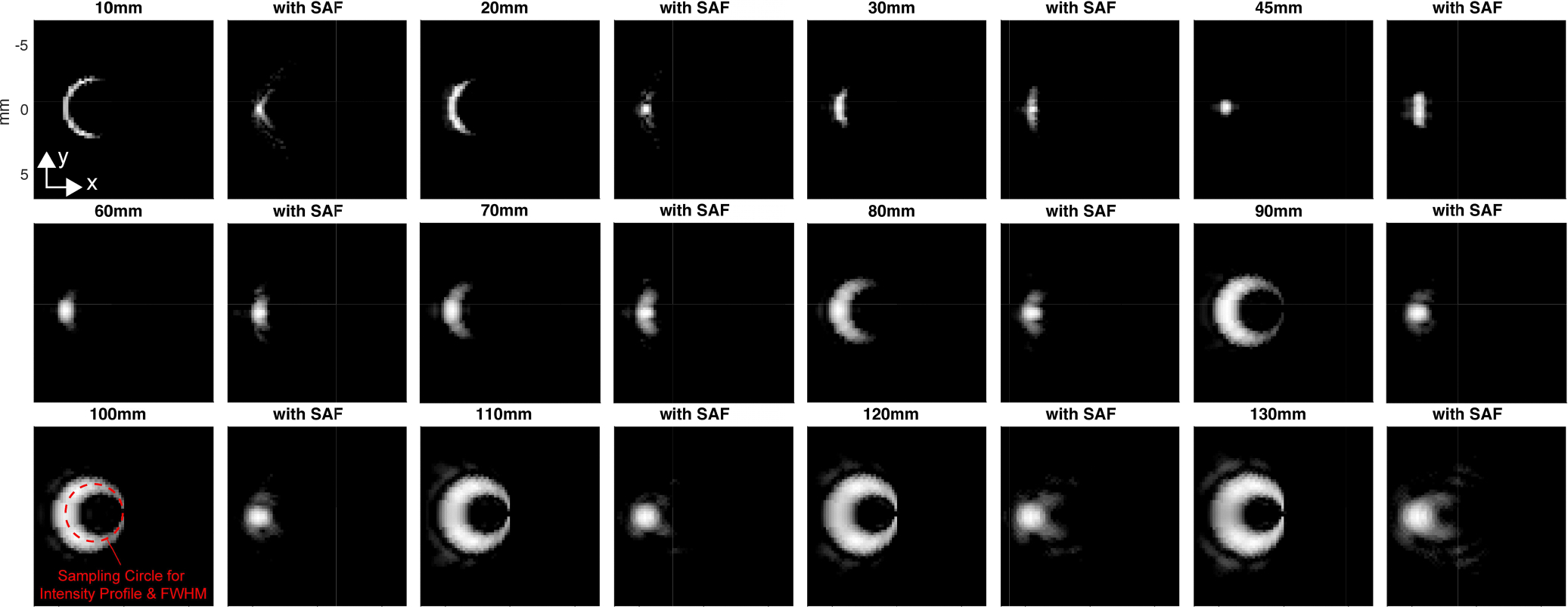
MIP images for the point target simulation study (The dynamic range is 20 dB) For each depth, the left and right images represent without and with the SAF algorithm, respectively.

To evaluate the focusing performance of the SAF algorithm, a scatter is placed at several three-dimensional positions. The fluctuating parameters here are the depth and the distance from the rotation center. As represented in the MIP images shown in Figure 6, where the distance between the scatter and the rotation center is 4 mm, it is confirmed that the point target is distorted due to the rotational motion and the amount of distortion gets greater as the target is placed farther from the elevational focal depth, which is around 45 mm in this case. By displaying the data in MIP, the 3D data is compressed into 2D by projecting the maximum value of pixels along the projection path. The results with the SAF algorithm show that the distortion is suppressed, which can be observed.

**FIGURE 7.**
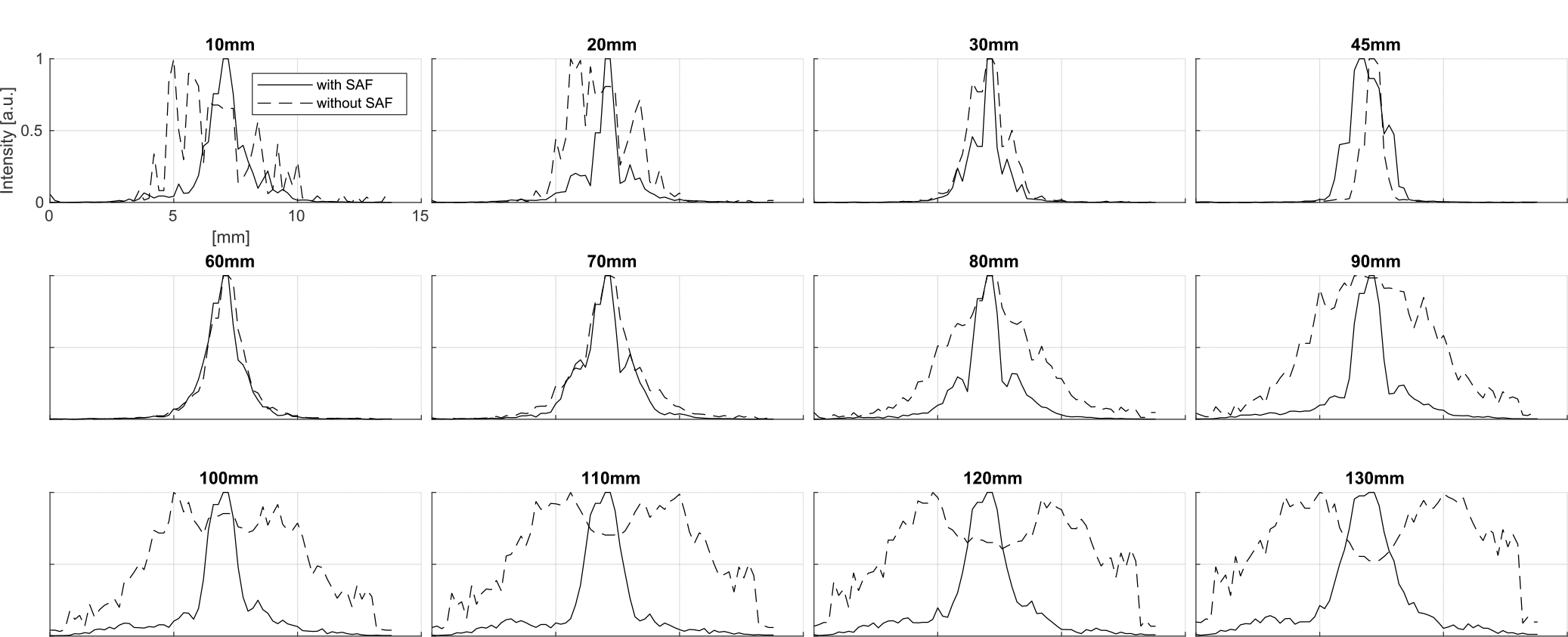
Intensity profile corresponding to the simulated MIP images with/without the SAF algorithm (Figure 6). The profiles are sampled along the circular line to take into account the rotational motion.

The corresponding beam profiles (Figure 7) indicate that the SAF algorithm also improves the resolution in the elevational direction except for some depths around 45 mm, the expected focal depth. It is noteworthy that although the actual locations do not have the peak intensity in the cases of 100 – 130 mm depths, the SAF algorithm focuses on the distorted signal and identifies the actual signal source location, which demonstrates its focusing capability beyond improving resolution.

**FIGURE 8.**
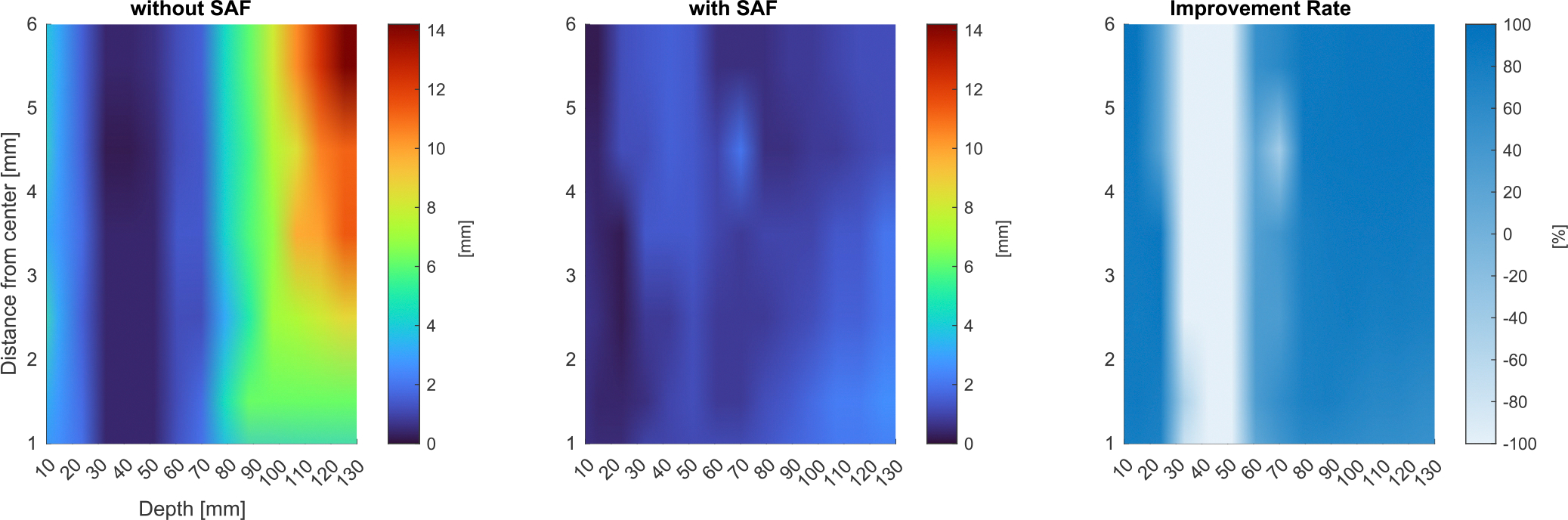
Parametric comprehensive simulation study of FWHM. (Left) Without SAF, (Center) With SAF, (Right) Improved Rate after SAF. Each FWHM is calculated by sampling the circular line to take into account the rotational motion.

For a more comprehensive analysis, the same analysis was performed for other distances from the rotation center and the results are summarized with the improvement rate in color (Figure 8). It is observed that the SAF algorithm mostly improves the FWHM except for the depths around the focal depth. In this region, the signal is already focused to some extent.

#### 2) CYST TARGET

**FIGURE 10.**
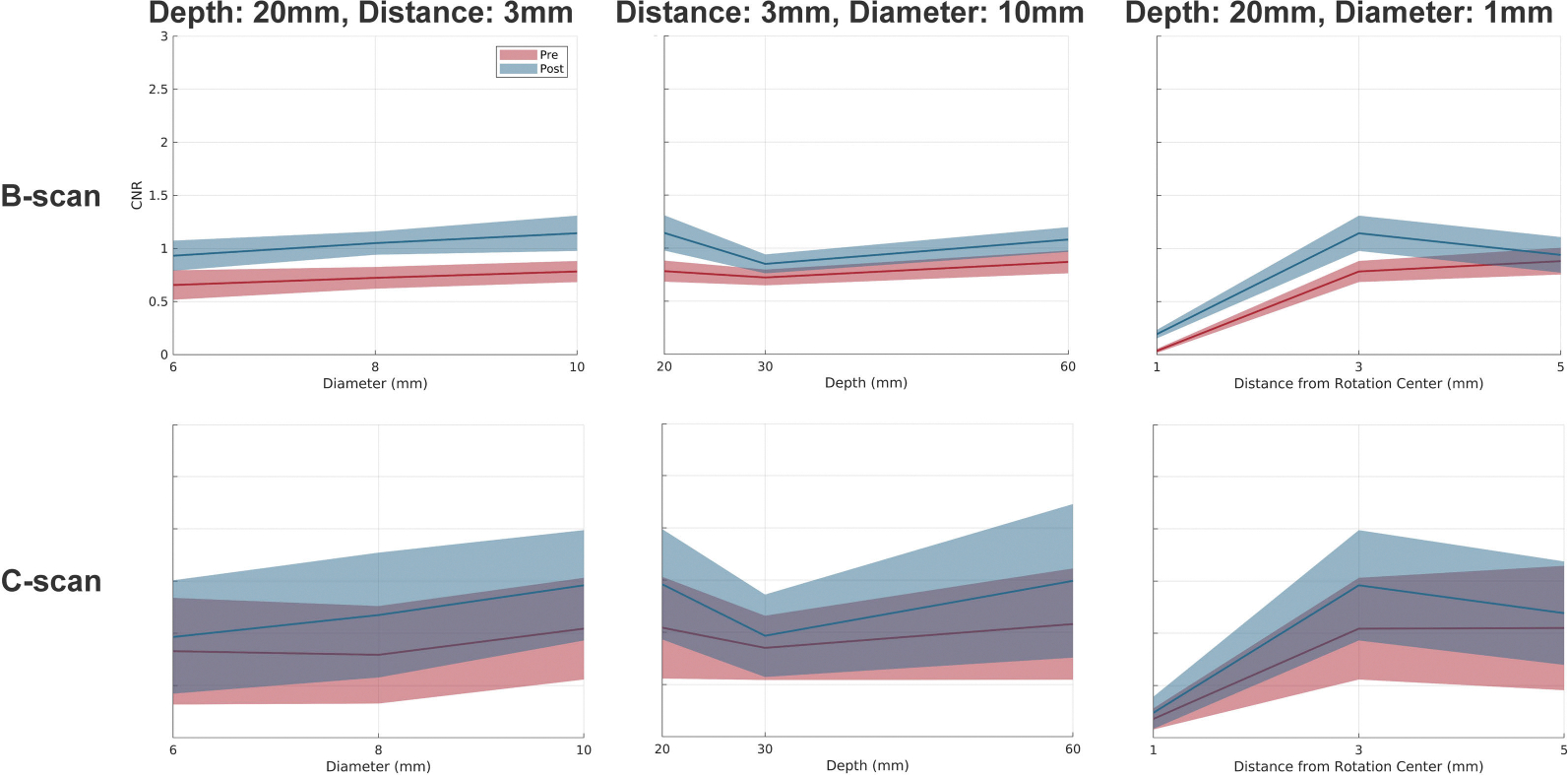
Comprehensive simulation study of the cyst imaging performance. The CNR is calculated for each condition and the average and the standard deviation are shown. The results are shown for the B-scan and the C-scan. (Left): Diameter of cyst as a parameter, (Center): Depth of cyst as a parameter, (Right): Distance from the rotation center as a parameter. (“Distance” is the abbriviation of “Distance from the rotation center”.)

The algorithm’s cyst imaging performance was evaluated in the simulation environment using CNR. For a comprehensive evaluation, several cyst-related conditions were considered: the cyst size (Diameter: 6, 8, and 10 mm), the cyst depth (20, 30, and 60 mm), and the distance from the rotation center (1, 3, and 5 mm). For each condition, the B-scan and the C-scan are generated. Since using the random scatter conditions, 10 trials were conducted for each conditions and the average and the standard deviation of CNR were calculated. The results are summarized in Figure 10. The results show that the SAF algorithm improves the CNR at least in all the conditions shown in Figure 10 including the B-scan and the C-scan. This improvement seems to be primarily attributed to the improved elevational resolution, which is demonstrated in Figure 8.

### B. EXPERIMENTAL STUDY

#### 1) POINT TARGET

**FIGURE 11.**
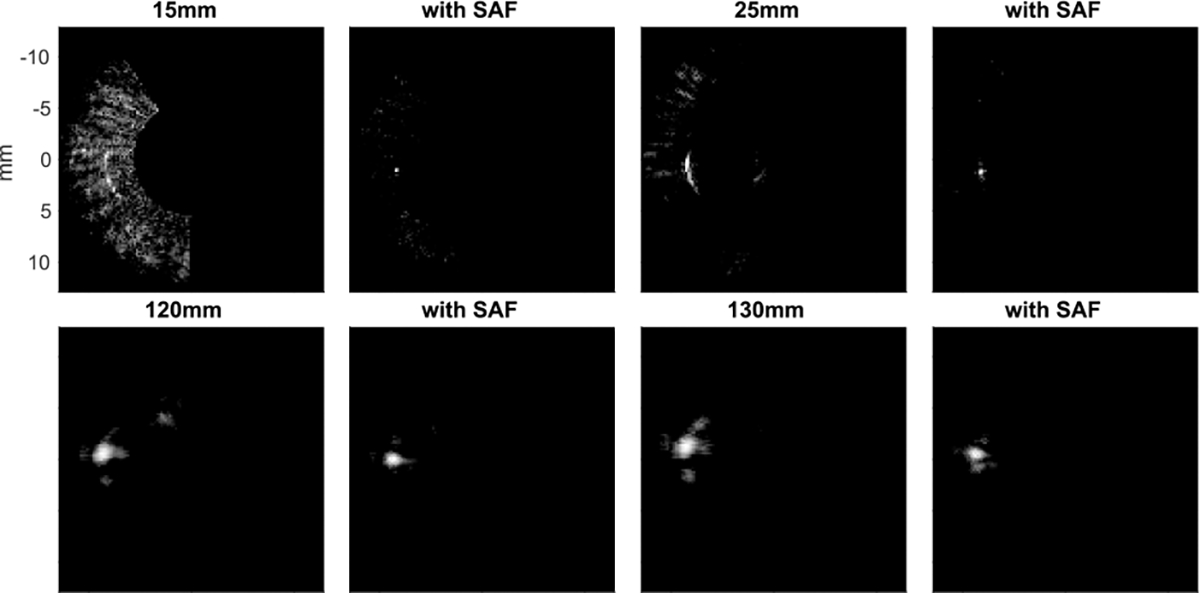
MIP images of the experiment results with point target placed at the depths of 15, 25, 120 and 130 mm. For each depth, the left and right images represent without and with the SAF algorithm, respectively (The dynamic range is 10dB ). Note that each image was independently rotated so that the target is located on the left and at the vertical center for the interest of image processing.

The resolution-related performance of the SAF algorithm was also evaluated in the experimental setup. The sphere bead as a target was placed at depths of 15, 25, 120, and 130 mm. The imaging device was rotated by 180 degrees with the step of 1 degree with plane waves transmitted. The MIP images were generated both for the cases with/without the SAF algorithm (Figure 11). Since it was observed that the elevational focus of the probe was effective until around 70 mm in depth, the focal depth for the SAF algorithm was set to 45 mm and 70 mm for shallower and deeper regions, respectively. The overall trend is consistent with the simulation results shown in Figure 6, and the SAF algorithm successfully suppresses the distortion along the elevation directions.

**FIGURE 12.**
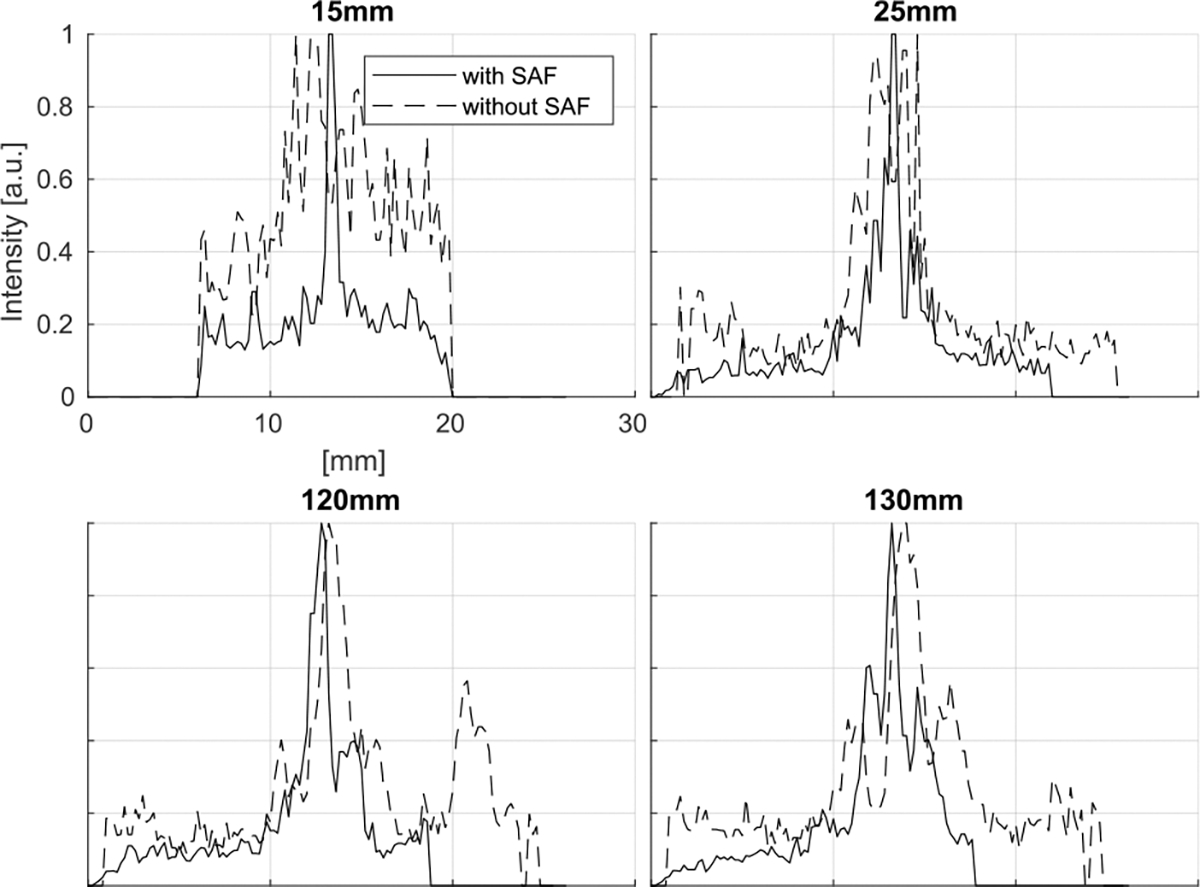
Intensity profile corresponding to the simulated MIP images with/without the SAF algorithm (Figure 11). The profiles are sampled along the circular line to take into account the rotational motion.

The corresponding intensity profile is displayed in Figure 12. As summarized in Table 2, the FWHM is improved by the SAF algorithm in all the cases.

#### 2) BREAST PHANTOM

**FIGURE 13.**
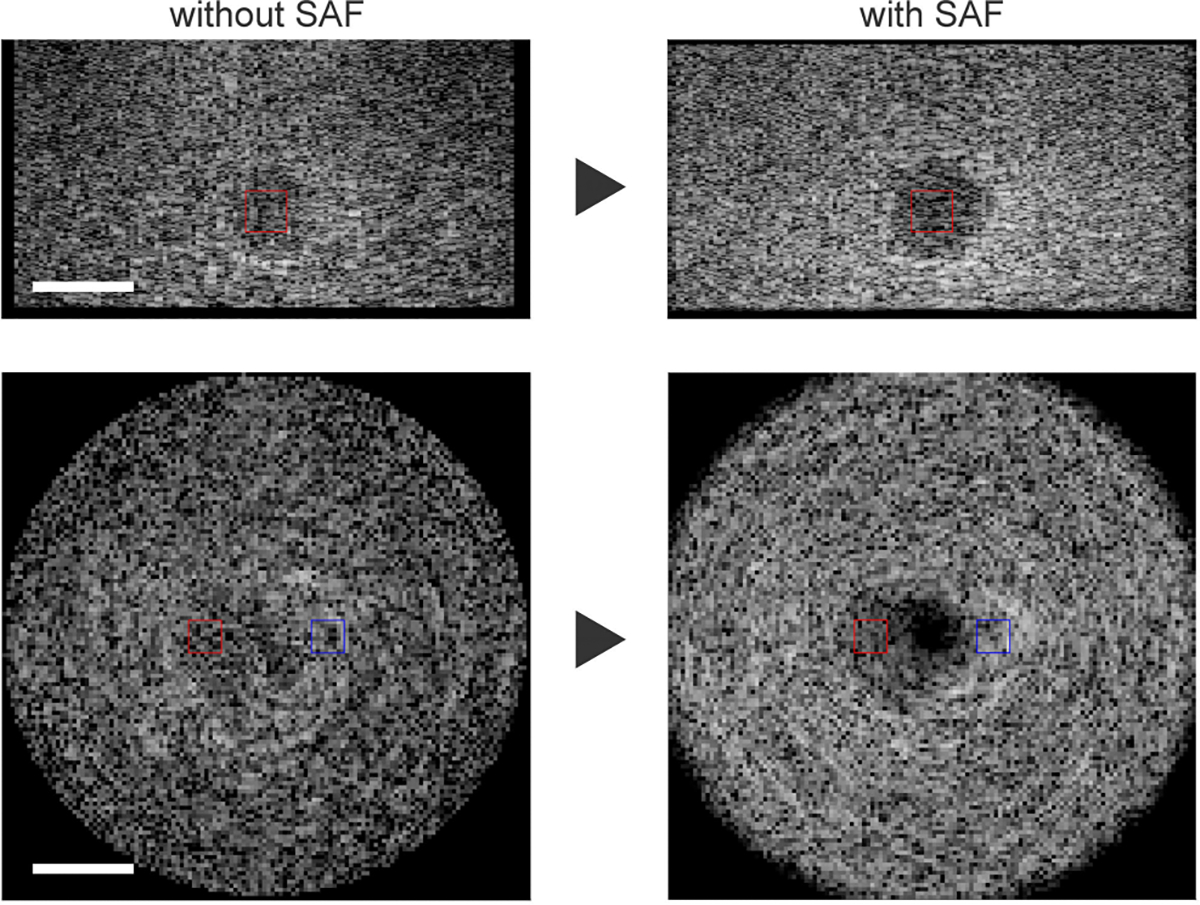
Cyst imaging in the breast phantom (Figure 5). (Top) B-scan, (Bottom) C-scan, (Left) without SAF, (Right) with SAF (The dynamic range is 35 dB. Scale bar: 5 mm. Window for CNR: Red: Target, Blue: Background.)

The performance of the SAF algorithm for cyst imaging is experimentally evaluated using the breast phantom which contains simulated cysts. One of the cysts underwent the rotational 3D US imaging with focused-multi-angled waves transmitted and the SAF algorithm was applied to the data, then both B-scan and C-scan images were generated (Figure 13). The imaged cyst with SAF provides higher CNR compared to the one without SAF in both B-scan and C-scan cases. The overall trends are aligned with the simulation results and it can be claimed that the developed SAF algorithm is effective for cyst imaging.

## IV. DISCUSSIONS

In this study, the SAF algorithm for rotational 3D US imaging has been proposed to enhance image resolution and contrast. In addition, we aimed to make the algorithm compatible with the highly accessible US data, which is the in-plane beam-focused RF data. The performance of the algorithm was evaluated using both simulation and experimental settings by assuming one specific transducer as an example.

Based on the results, several benefits have been found and can be summarized as follows. First, it has been demonstrated that the SAF algorithm has the potential to improve image quality in terms of resolution and contrast. The greatest contrast enhancement observed in this study was almost 200 %. Also, it is noteworthy that these improvements were achieved without any additional hardware except for devices necessary for rotational 3D US imaging; moreover, any type of existing transducer is expected to work with the algorithm. This enhances the accessibility of this technique in both research and clinical environments. Lastly, the SAF algorithm is expected to reduce the scanning time for 3D US imaging compared to the case without this algorithm because the algorithm would widen the effective depth range of the 3D US by improving the resolution and contrast of the images; otherwise, the data from the depth far from the focal depth would be discarded due to the poor image resolution and contrast.

This study implies several limitations and challenges of this SAF algorithm. For example, the elevational beam profile of a transducer needs to be well-investigated to apply the SAF algorithm effectively. In fact, the results show that the algorithm can worsen the resolution around the focal depth. Given this limitation, the transducers whose elevational focal depth is clearly specified would be more suitable for this SAF algorithm. Therefore, testing the algorithm using only one single transducer can be considered one of the limitations of this study. The deterioration of numerical values due to the algorithm was observed not only in the vicinity of the focal depth but also in other instances. Specifically, in the verification shown in Figure 10, when the cyst depth was set to 80 mm, a slight degradation in CNR was observed after applying SAF. Currently, this phenomenon is thought to be attributable to factors such as the initially wide lateral distribution of scatter images at 80 mm depth, even in B-mode imaging (as can be confirmed in Figure 6). Another limitation is that the algorithm is currently not optimized for real-time imaging. Although we prioritized image quality over frame rate in this specific study as discussed in Introduction, the algorithm has room to improve in terms of frame rate. In order to make the algorithm available for real-time imaging, the algorithm should be further optimized, and fast programming languages such as C++ or GPU acceleration should be considered [24]. Other limitations include the lack of sensitivity analysis regarding the scanning sequence-related parameters. For example, the effect of the number of imaging slices on the performance of the SAF algorithm should be investigated, which can reduce the number of slices resulting in shorter scanning and computation time.

## V. CONCLUSION

In this study, the SAF algorithm for rotational 3D US imaging enhancing image resolution and contrast has been proposed and implemented. The performance evaluation using the simulations and the phantoms shows that the SAF algorithm can improve the resolution and contrast of the US images. Given that this algorithm assumes in-plane-focused US images, it is expected that the SAF algorithm for rotational 3D US will become more accessible in both research and clinical settings. Our future work involves the further evaluation of this algorithm using different probes and targets.

## Figures and Tables

**FIGURE 9.**
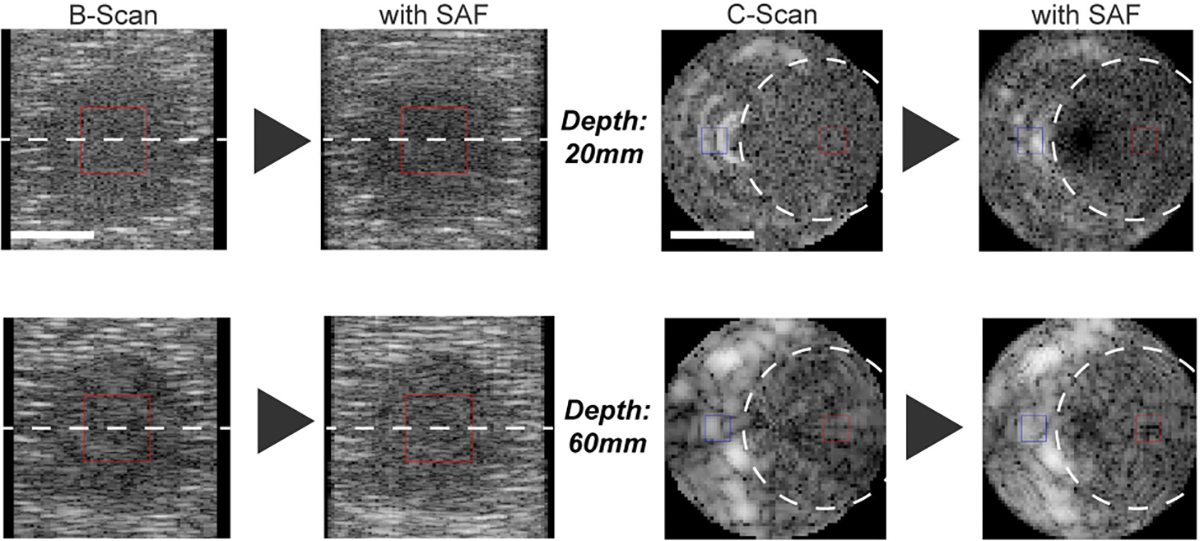
Simulated cyst phantoms placed at 20 and 60 mm in depth (Left: B-scan, Right: C-scan, Dynamic Range: 60dB. Scale Bar: 5mm. Window for CNR: Red: Target, Blue: Background).

**TABLE 1.** Transducer parameters with speed of sound.

Parameter	Notation	Value	Unit

Speed of sound	*c*	1490	m/s
Center frequency	*f*_0_	10	MHz
Sampling frequency	*f_s_*	40	MHz
Number of elements	*N*	68	-
Element pitch	*p*	0.2	mm
Element width	*w*	0.15	mm
Element height	*h*	8	mm
Curvature for elevational focus	*R*	45	mm

**TABLE 2.** FWHM in Experiment (Figure 12).

Depth (mm)	FWHM (mm)	
	without SAF	with SAF

15	11.0	0.4
25	3.4	0.8
120	8.2	1.0
130	3.2	2.8

**TABLE 3.** CNR in Breast Phantom (Figure 13) (Approx. depth 27 mm).

Type	without SAF	with SAF	Improve (%)

B-scan	0.29	1.09	274.7
C-scan	0.53	1.01	90.6
